# Bilateral Optic Nerve Sheath Meningioma with Intracanalicular and Intracranial Component in a 25-year-old Saudi Patient

**DOI:** 10.4103/0974-9233.51990

**Published:** 2008

**Authors:** Maha A. Badr, Sahar M. Elkhamary, Samira Al Sabbagh, Abdulsalam Al Turjoman

**Affiliations:** From Neuro-ophthalmology Division, King Khaled Eye Specialist Hospital, Riyadh, Saudi Arabia; * From Diagostic Imaging Department, King Khaled Eye Specialist Hospital, Riyadh, Saudi Arabia

**Keywords:** meningioma, optic nerve tumor, optic nerve sheath meningioma, MRI

## Abstract

Bilateral optic nerve sheath meningioma is rare. A meningioma is a benign neoplastic lesion from meningothelial cells of the meninges. They usually involve the intracanalicular portion of the optic nerve but may extend into the optic canal and through it to occupy the intracranial space. We present a case of 25-year-old Saudi female with bilateral optic nerve sheath meningioma. The diagnosis was delayed more than six years from initial symptoms.

Bilateral optic nerve sheath meningioma is rare. A meningioma is a benign neoplastic lesion from meningothelial cells of the arachnoid. They usually involve the optic nerve in the orbit but may extend into the optic canal and through it to occupy the intracranial space. Optic nerve sheath meningiomas are unilateral on most of the cases.^1^,^2^ The usual presentation is a slowly progressive visual loss and optic atrophy or disc swelling. At presentation opticociliary shunt vessels are found in the optic disc in about 20% of the cases.^3^,^4^ Bilateral optic nerve sheath meningioma leads to visual deterioration in the both eyes - one preceding the other. The fact that they have relative intact visual acuity may obscure the present of significant optic neuropathy. The diagnosis of optic nerve sheath meningioma is based on the appropriate clinical picture supported by appropriate neuroimaging.^4^,^5^

## Case Report

**Figure 1:**
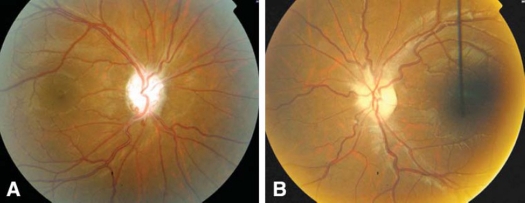
**(A)** Right eye shows pale disc with retinochoroidal collateral; **(B)** Left eye shows very mild temporal pallor.

A 25-year-old Saudi female was presented to Neuro-ophthalmology clinic at King Khaled Eye Specialist Hospital, Riyadh, complaining of a gradual vision decrease in the right eye since seven years. During this period, she has been seen in many hospitals and treated with different eye drops. There was no previous history of eye pain, eye trauma or any medical disorders. Recently, she complained of marked deterioration of vision in the right eye with a history of mild intermittent headache. On examination, the best corrected visual acuity (BCVA) was 2/200 in the right eye and 20/25 in the left eye. Extraocular eye movement was normal, saccade and pursuit was normal with no nystagmus. Cranial nerve examination was also found to be normal. The pupillary reaction was sluggish to direct light with relative afferent papillary defect in the right eye and normal in the left eye. Slit lamp examination of the anterior segment was normal in the both eyes. The fundscopic examination showed optic atrophy with retinochoroidal collateral (optociliary shunt) in the right eye and the left optic disc showed mild temporal pallor (Fig 1). Humphrey visual field was done and it showed marked generalized constriction of the visual field in the right eye and mild peripheral visual field depression in the left eye.

**Figure 2:**
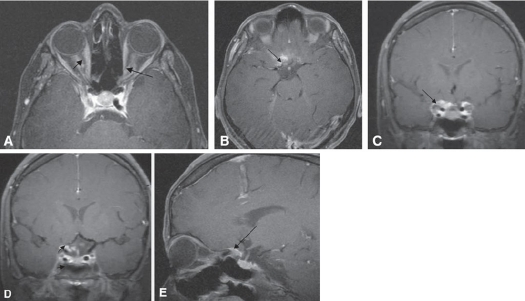
Post contrast T1-weighted MR image axial MRI with fat saturation showed Tubular growth pattern of bilateral optic nerve sheath meningioma as diffuse enhancement along the length of the left Intraconal optic nerve sheath (black arrows). Optic nerve is seen as central linear hypointensity in comparison to enhanced meningioma on either side, producing the tramtrack sign **(A)**. Axial and coronal T1-weighted, contrast-enhanced images (**B** and **C**) showed diffuse enlargement and enhancement along the RT intracanalicular (white arrows) optic nerve sheath with en-plaque growth along the walls of the sulcus chiasmaticus, giving a “rose thorn” appearance (black long arrow). Contrast-enhanced coronal and sagittal T1-weighted MR image, with fat suppression through the optic nerve in the midorbit, illustrates enhancing mass affecting the RT planumsphenoidale and inferior orbital fissures with en-plaque growth of tumor within the right optic nerve sheath. The left side meningioma, the tumor was limited to the canal and the immediately adjacent walls of the sulcus chiasmaticus and orbital apex (**D** and **E**).

Post contrast T1-weighted MR image axial MRI with fat saturation (Fig 2 A–D) showed tubular growth pattern of bilateral optic nerve sheath meningioma as diffuse enhancement along the length of the left intraconal optic nerve sheath (black arrows). Optic nerve is seen as central linear hypointensity in comparison to enhanced meningioma on either side, producing the tram-track sign with extensive enhancement along the RT intracanalicular optic nerve sheath (white arrows) with en-plaque growth along the walls of the sulcus chiasmaticus, giving a “rose thorn” appearance(black long arrow). Contrast-enhanced coronal and sagittal T1-weighted MR image, with fat suppression through the optic nerve in the midorbit (Fig 2 D–E), illustrates enhancing mass affecting the RT planumsphenoidale and inferior orbital fissures with en-plaque growth of tumor within the right optic nerve sheath. The left side meningioma is limited to the sulcus chiasmaticus and orbital apex.

**Figure 3 (A-B):**
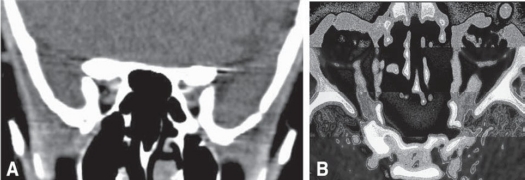
Non-contrast CT images, coronal and axial CT scan showed linear calcification of the nerve suggestive of a tram track appearance with mild hyperostosis.

Non-contrast coronal and axial CT images (Fig 3 A–B) clearly detect the linear calcification along the optic nerve sheath - suggestive of a tram track appearance with mild hyperostosis. CT and MRI confirmed the diagnosis of bilateral optic nerve sheath meningioma.

## Discussion

Optic nerve sheath meningiomas are primary tumors that arise from meningothelial cells of the meninges. Although they may be present at any age, they are predominately seen in the middle-aged females.^6^,^7^ They usually involve the optic nerve in the orbit but may extend into the optic canal and through it to occupy the intracranial space. Slow, progressive visual loss, which may be unilateral or bilateral, is the only symptom of this disorder. Patients usually have good visual acuity due to preservation of central vision. Good central vision so often masquerade the early diagnosis - especially if complete ophthalmic and neuro-ophthalmic evaluation not completed including looking for signs and symptoms of visual dysfunction: decrease visual acuity, visual field defect, color vision impairment pupillary test for presence of asymmetrical or absence of afferent pupillary defect, visual field examination and careful optic disc evaluation for asymmetry of disc color or pallor. In late stages, the optic disc might show characteristic vasculature optociliary shunt vessels changes in about 20% of cases.^8^,^9^ Optic nerve sheath meningioma is usually unilateral. Bilateral cases are rare and represent 5% of reported cases.^10^–^12^ Unilateral optic nerve sheath meningiomas are thought to be more common in patients with neurofibromatosis.^1^,^2^,^4^ Bilateral cases are usually isolated. Cunliffe IA, et al and Martina et al^13^ reported cases of bilateral optic nerve sheath meningioma in patients with neurofibromatosis Type II.^6^,^14^

Radiological diagnosis of optic canal meningioma is a crucial one and it is not always straightforward. Radiologically, these tumors may be extremely small despite causing significant symptoms and can easily be overlooked on routine imaging protocols.^15^

The ideal imaging protocols for the diagnosis should demonstrate the orbital extension of the tumor relied on a combination of contrast enhancement, high spatial resolution fat suppression sequences. The use of multiplanar image reconstruction is not essential for the demonstration of disease but it is helpful in appreciating the topography and extent of individual lesions.^16^

The radiographic growth patterns of optic nerve sheath meningiomas are variable. Meningiomas are typically en plaque form and grow linearly along the nerve sheath. Three distinct growth patterns have been ascribed to optic nerve sheath meningiomas.^14^,^16^ These include tubular enlargement, in which there is uniform expansion in the cross-sectional area of the nerve sheath which is subdivided into diffuse expansion, apical expansion towards the orbital apex, or anterior nerve expansion towards the globe. The second growth pattern is the fusiform pattern, in which there is a spindle-shaped increase in the cross-sectional area; with tapers at the proximal and distal ends. This type is most likely to be confused with optic nerve glioma. The last one is the excrescent globular growth patterns - caused by exophytic outgrowth emanating from or attached to the sheath.

In this study, axial CT images with thin section (1.5-mm), reveal regular thickening of the nerve sheath meninges with moderate to marked homogenous enhancement after intravenous contrast infusion (Figs 3 A–B). This is typical with the appearance of a “tram track.” of the hyperdense enhancement of the meninges surrounding a hypodense non-enhancing nerve. Similarly, on non-contrast CT images, linear calcification of the nerve is also suggestive of a tram track.^17^,^18^

Mafee et al^15^ described that the optic nerve itself may appear normal or smaller in diameter within an area of thickened meninges giving a bull's eye appearance on coronal images and a tram track appearance on axial images.^18^ The smaller nerve size is the result of circumferential compression or atrophy and is a useful differential point with other pathology. It causes an intrinsically expanded nerve as more commonly seen in optic nerve glioma or other inflammatory lesions. While in optic nerve glioma, there is no bull's eye appearance on coronal section, but a sagittal kinking is often seen.

In our case, we agree with Mafee study^15^ who reported that bilateral optic nerve sheath meningiomas although are rare, 65% of reported cases are intracanalicular.^18^ With a discrete tumor nodule and en-plaque growth with a “rose thorn” appearance with the calcification may be demonstrated in 20-50% of cases with optic nerve sheath meningiomas (Fig 2 B–D).

The exact knowledge of bony involvement in meningiomas is imperative because unresected meningiomatosis bone is one of the important factors that may contribute to recurrence. At CT, osseous changes may be present in the region of the optic nerve canal, such as hyperostosis and occasionally bone erosion.^14^,^16^

MRI remains the procedure of choice for diagnosis of juxtaorbital meningiomas although it is less sensitive than CT in the recognition of calcification. Currently high field strength (≥1.5 tesla) using T1-weighted gadolinium contrast fat suppression images. On most MRI pulse sequences, juxtaorbital meningiomas are typically isointense or slightly hypointense to brain and optic nerve tissue on T1-weighted images and hyperintense on T2-weighted images but may also be hypointense. Gadolinium-enhanced fat-suppression, T1-weighted pulse sequences allow visualization of meningiomas as a localized or tubular enlargement with significant contrast enhancement as well as detect the subtle meningeal enhancement and minimal parenchymal edema (Fig 2B).

The tram-track sign is most evident on fat-suppressed T1-weighted magnetic resonance (MR) images of the orbit. On these images, the non-enhancing optic nerve appears as a negative defect in relation to the surrounding enhancement in the area of the optic nerve sheath on either side. The corresponding finding on coronal images is a doughnut configuration.^14^ Though it is less common, the tram-track sign is non-specific and can be seen in conjunction with other orbital diseases, including orbital pseudotumor, perioptic neuritis, sarcoidosis, leukemia, lymphoma, metastases, perioptic hemorrhage, and Erdheim-Chester disease. In addition, enhancement of the periphery of an optic nerve, which is not enlarged, may be a normal finding and probably represents normal dural enhancement.^14^,^17^,^19^,^20^ Radiological diagnosis of optic canal meningioma is a crucial one and it is not always straightforward.^8^,^9^,^21^–^23^

In summary the diagnosis of optic nerve sheath meningioma is based on the appropriate clinical picture supported by appropriate neuroimaging. Proper and full ophthalmic evaluation including pupillary function, color vision and visual field are mandatory and will help in early diagnosis. Imaging of patients with suspected optic neuropathy should certainly be performed in cases in which the clinical presentation is atypical or where progressive visual loss continues despite treatment, and MR is clearly superior to CT in this situation. This requires coverage of the entire optic nerve from the globe to the optic tracts and must employ adequate spatial resolution and required fat suppression in the orbit, in combination with contrast enhancement to demonstrate blood vessels and any pathologic process.

## References

[1] Karp LA, Zimmerman LE, Borit A, Spencer W (1974). Primary intraorbital meningiomas. Arch Ophthalmol.

[2] Wilson WB (1981). Meningiomas of the anterior visual svstem. Surn Ophthalmol.

[3] Sibony P, Krauss HR, Kennerdell JS (1984). Optic nerve sheath meningiomas: Clinical manifestations. Ophthalmology.

[4] Jakobiec FA, Depot MJ, Kennerdell JS (1984). Combined clinical and computed tomographic diagnosis of orbital glioma and meningioma. Ophthalmology.

[5] Sarkies NJC (1987). Optic nerve sheath meningioma: diagnostic features and therapeutic alternatives. Eye.

[6] Cunliffe IA, Moffat DA, Hardy DJ, Moore AT (1992). Bilateral optic nerve sheath meningioma in a patient with neurofibromatosis type 2. Br J Ophthalmol.

[7] Saeed P, Rootman J, Nugent RA (2003). Optic nerve sheath meningiomas. Ophthalmology.

[8] Hollenhorst RW, Hollhorst RW, MacCarty CS (1978). Visual prognosis of optic nerve sheath meningioma producing shunt vessels on the optic disc. Mayo Clin PROC.

[9] Spencer WH (1972). Primary neoplasms of the optic nerve and itssheaths: clinical features and current concepts of pathogenetic mechanisms. Trans Am Ophthalmol Soc.

[10] Craig WM, Gogela LJ (1949). Intraorbital meningiomas: A clinicopathological study. Am J Ophthalmol.

[11] Tarbe JD, Glaser JS, Post JD (1978). Bilateral optic canal meningiomas: A case report. Neurosurgery.

[12] Ortiz O, Schochet SS, Kotzan JM, Kostick D (1996). Radiologic-pathologic correlation: meningioma of the optic nerve sheath. AJNR Am J Neuroradiol.

[13] Martina MB, Werner WW, Eugen B, Klara L (2006). Optic nervesheath meningiomas in patients with neurofibromatosis Type 2. Arch Ophthalmol.

[14] Hart WM, Burde RM, Klingele TG (1980). Bilateral optic sheath meningiomas. Arch Ophthalmol.

[15] Mafee MF, Goodwin J, Dorodi S (1999). Optic nerve sheath meningiomas: role of MR imaging. Radiol Clin North Am.

[16] Jackson A, Sheppard S, Johnson AC (1999). Combined fat- and water-suppressed MR imaging of orbital tumors. AJNR Am J Neuroradiol.

[17] Uday S (2003). Kanamalla: The optic nerve tram-track sign. Radiology.

[18] Castel A, Boschi A, Renard L, De Potter P (2000). Optic nerve sheath meningiomas: clinical features, functional prognosis and controversial treatment. Bull Soc Belge Ophtalmol.

[19] Jackson A, Sheppard S, Johnson AC (1999). Combined fat- and water-suppressed MR imaging of orbital tumors. AJNR Am J Neuroradiol.

[20] Egan RA, Lessell S (2002). A contribution to the natural history of optic nerve sheath meningiomas. Arch Ophthalmol.

[21] Vaphiades MS (2001). Disk edema and cranial MRI optic nerve enhancement: how long is too long?. Surv Ophthalmol.

[22] Jackson A, Sheppard S, Laitt RD (1998). Optic neuritis: MR imaging with combined fat- and water-suppression techniques. Radiology.

[23] Mafee MF, Goodwin J, Dorodi S (1999). Optic nerve sheath meningiomas:role of MR imaging. Radiol Clin North Am.

